# Cultured Autologous Corneal Epithelia for the Treatment of Unilateral Limbal Stem Cell Deficiency: A Case Series of 15 Patients

**DOI:** 10.3390/biomedicines10081958

**Published:** 2022-08-12

**Authors:** Louis-Philippe Guérin, Danielle Larouche, Mohib W. Morcos, Anne Faucher, François A. Auger, Bartha M. Knoppers, Ralph Kyrillos, Richard Bazin, Lucie Germain

**Affiliations:** 1 Centre de Recherche en Organogénèse Expérimentale de l’Université Laval/LOEX, Quebec, QC G1J 1Z4, Canada; 2 Centre de Recherche du CHU de Québec-Université Laval, Quebec, QC G1J 1Z4, Canada; 3 Département de Chirurgie Faculté de Médecine, Université Laval, Quebec, QC G1V 0A6, Canada; 4 Département d’Ophtalmologie, Faculté de Médecine, Université Laval, Quebec, QC G1V 0A6, Canada; 5 Service d’Ophtalmologie, CHU de Québec-Université Laval, Québec, QC G1S 4L8, Canada; 6 CUO-Recherche/LOEX, Québec, QC G1S 4L8, Canada; 7 Service d’Ophtalmologie, Université de Sherbrooke, Sherbrooke, QC J1G 2E8, Canada; 8 Service de Pathologie, CHU de Québec-Université Laval, Quebec, QC G1S 4L8, Canada; 9 Département de Pathologie, CISSS de l’Outaouais, Hôpital de Hull, Gatineau, QC J8Y 1W7, Canada; 10 Centre of Genomics and Policy, Faculty of Medicine, Department of Human Genetics, McGill University, Montreal, QC H3A 0G1, Canada

**Keywords:** cultivated limbal epithelial transplant CLET, cornea, stem cells, limbal stem cell deficiency/LSCD, eye

## Abstract

Damage to limbal epithelial stem cells can lead to limbal stem cell deficiency (LSCD). Current autologous treatment procedures for unilateral LSCD bear a significant risk of inducing LSCD in the donor eye. This complication can be avoided by grafting a stem cell containing cultured autologous corneal epithelium (CACE). The primary objective of this study was to demonstrate the safety of CACE grafted on eyes with LSCD. The secondary objective was to assess the efficacy of a CACE graft in restoring a self-renewing corneal surface with adequate anatomic structures, as well as improving the best corrected visual acuity (BCVA). Fifteen patients were grafted with a CACE on a fibrin gel produced from a 3 mm^2^ limbal biopsy harvested from the donor eye. Data were collected at baseline and after grafting. Follow-ups from 1 to 5 years were conducted. No major adverse events related to the CACE graft were observed. For every visit, an anatomic score based on corneal opacity as well as central vascularization and a functional score based on BCVA were determined. Safety was demonstrated by the low occurrence of complications. Anatomical (93%) and functional (47%) results are promising for improving vision in LSCD patients. Combined functional success and partial success rates with inclusion of BCVA were 53% [CI95: 27–79%] one year after CACE grafting. At the last follow-up, 87% [CI95: 60–98%] of the patients had attained corneal clarity. The outcomes demonstrate the safety of our technique and are promising regarding the efficacy of CACE in these patients.

## 1. Introduction

Vision results from the successful passage of light to the retina. To reach the retina, light must go through a transparent cornea. This depends on the appropriate structure and function of the corneal epithelium, stroma and endothelium. The corneal epithelium is composed of 5–7 layers of stratified and non-keratinized squamous epithelial cells lying on a basement membrane [1]. These cells proliferate and migrate centripetally from the periphery to the center of the cornea via the basal layer and then from the deeper layer to the superficial layers to replace the epithelial cells desquamated from the corneal surface. This ensures homeostasis according to the ‘X, Y, Z hypothesis’ proposed by Thoft and Friend [2]. This self-renewing mechanism is made possible by the undifferentiated stem cells located in Vogt’s palisades at the corneal limbus [3]. Many diseases can affect corneal transparency; among these, stem cell deficiency might be one of the most difficult to treat.

Damage to limbal epithelial stem cells or to their microenvironment can lead to limbal stem cell deficiency (LSCD). The cause can either be primary, such as in congenital aniridia, congenital epidermal dysplasia or dyskeratosis congenita, or secondary, resulting from thermal or chemical burns, mucous membrane pemphigoid, Stevens-Johnson syndrome, graft vs. host disease, chronic limbitis or iatrogenic injury from surgeries, radiation or chemotherapy. Other causes include neurotrophic keratitis, extensive microbial infection, severe prolonged bullous keratopathy, tumor growth and treatment, chronic use of ophthalmic drops and long-term contact lens wear, and idiopathic etiologies [4].

The clinical presentation of LSCD depends on the severity of the underlying disease. Manifestations include decreased visual acuity, inflammation of the ocular surface, chronic pain, persistent erosions, thinning, scarring, conjunctivalization and neovascularization of the corneal surface [5,6]. Partial LSCD can be managed with medical treatment by optimizing ocular surface health. This can be attained with eye lubrication and topical corticosteroids. However, severe cases of LSCD often require surgical treatment, especially when the visual axis is involved [6,7].

In unilateral LSCD, the healthy contralateral eye can provide the source of limbal stem cells required for transplantation. The most commonly performed procedure in unilateral LSCD is the conjunctival limbal autograft (CLAU), in which an opposing pair of two clock-hour-long conjunctival-limbal biopsies are harvested from the healthy eye to be grafted to the affected eye. The main complication with this technique is the risk of inducing iatrogenic LSCD in the healthy donor eye [8,9].

New methods have been developed to decrease the risks associated with CLAU. Cultivated limbal epithelial transplant (CLET) is a technique in which a small biopsy (approximately 2–3 mm^2^) is harvested in a healthy eye. Cells are then expanded ex vivo on a human amniotic membrane or on a fibrin carrier scaffold and grafted onto the affected eye. The goal is to provide a long-term solution to LSCD by transplanting a self-renewing limbal stem cell population that can maintain a clear corneal epithelium [10,11]. The main advantage of this approach is the very small size of the limbal biopsy (2–3 mm^2^) needed to initiate the culture, thus significantly decreasing the risk of injury to the donor eye. In 2010, the long-term success of autologous grafting of cultured corneal epithelial cells grown on fibrin gel for the treatment of a large cohort of LSCD patients has been published. The success of these grafts was correlated with the presence of stem cells in the cultured corneal substitutes [12]. Following the safety demonstration, Holoclar^®^ was approved for LSCD by the European regulatory agency for commercial use. This type of regenerative medicine has also been used mainly in small clinical trials for the treatment of patients suffering from LSCD in other countries [10,13,14,15]. 

Simple limbal epithelial transplant (SLET), another available option for treating LSCD, has emerged. It revolves around a single biopsy, similar in size to the one in CLET, that is divided into 8–15 smaller pieces evenly glued at the periphery of a human amniotic membrane covering the debrided diseased cornea. Studies have shown promising results and the technique does not require laboratory facilities for ex vivo culture [16,17,18]. However, immediate grafting does not allow quality control before transplantation.

In the mid-90s, our team began to develop CLET in Canada [19]. After 30 years of experience with cultured epithelial autografts for permanent skin regeneration in severely burned patients, we successfully demonstrated the effectiveness of limbal epithelial cells cultured on fibrin gel (referred to as cultured autologous corneal epithelium or CACE) for ocular surface reconstruction in animal models [20] and in vitro reconstructed human corneas [21]. The epithelial cells were also characterized in vitro with various markers, including keratins and delta-N p63, as well as on 3D human tissue-engineered cornea [21,22,23,24,25]. In 2011, we obtained approval from Health Canada’s Special Access Program to graft a patient with LSCD. This complex case was treated successfully [24]. In 2012, our team obtained approval from the Biologic and Radiopharmaceutical Drugs Directorate of Health Canada to treat 15 patients with LSCD using CACE as a first early-phase study aimed at evaluating the safety of CACE for treating LSCD and generating the first efficacy data. The present report describes our preliminary results using CACE to treat various causes of LSCD. Results of the anatomical score and best corrected visual acuity are presented at 1 year and follow-ups until 5 years for a subset of patients. 

## 2. Materials and Methods

### 2.1. Study Design

This interventional, monocentric, single-group assignment study was conducted on 15 patients treated at the “Centre Universitaire d’Ophtalmologie” (CUO) of the CHU de Québec-Université Laval. Initially, the sample size was determined at 15 patients based on the annual number of cases of unilateral LSCD treated at CUO, which is approximately 5 per year. No power calculation was performed because this first early phase trial in Canada aimed to focus on product safety evaluation.

### 2.2. Study Population

This study was reviewed and approved by the research ethics committee of the CHU de Quebec-Université Laval. Patients were prospectively recruited from the “Centre Universitaire d’Ophtalmologie” (CUO) du CHU de Québec-Université Laval between March 2013 and January 2018. Adults of all age groups who presented with unilateral LSCD were included. The principal exclusion criteria were bilateral LSCD, pregnancy, breastfeeding mothers, patients not being apt to consent or an allergy to Aprotinin or bovine proteins. Prior to enrollment, written consent was obtained from every patient.

### 2.3. Baseline Evaluation

**Figure 1:**
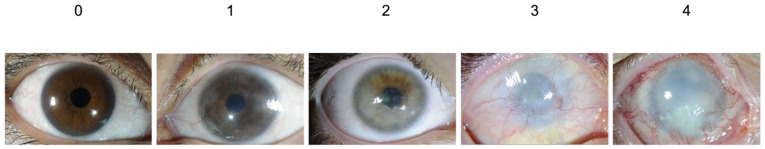
Corneal opacity score.

**Figure 2:**
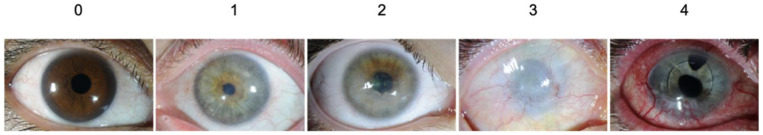
Corneal vascularization score.

The clinical assessment of the affected eye was based on slip lamp examination and scoring from 0 (normal) to 4 (highest severity level), typical symptoms associated with LSCD. Clinical findings, such as epithelial defect, determined with fluorescein staining; corneal opacity (Figure 1); peripheral vascularization; central vascularization (Figure 2); and integrity of the ocular surface determined by the presence of superficial punctate keratitis (SPK), contributed to the diagnosis and were used for the anatomical assessment.

The best corrected visual acuity (BCVA) was evaluated using a Snellen chart at a standard distance of 6 m. If visual acuity was not measurable using the Snellen chart, the following semi-quantitative scale was used: no light perception (NLP); light perception (LP); hand motion (HM); and counting fingers (CF). BCVA was used to evaluate the functional assessment.

A peripheral corneal biopsy was performed on 12 of the 15 patients to histologically confirm the diagnosis of LSCD (data not shown).

### 2.4. Histological Analysis

For histological analysis, biopsies of the affected eye were made at the limbus encompassing the conjunctival and corneal stroma. Tissue was embedded in Tissue-Tek OCT Compound (Sakura Finetek) and frozen in liquid nitrogen. Seven μm-thick sections were stained with hematoxylin eosin and periodic acid-Schiff. A pathologist ran the slides under a light microscope, interpreted the results, and then informed the surgeon of the LSCD diagnosis. Key findings in pathology were conjunctival metaplasia of corneal epithelium, in association with edema, congestion and vascular hypertrophy, with or without mild inflammation.

The CACE samples were fixed overnight in 3.7% formol and embedded in paraffin. Masson’s trichrome (Weigert’s hematoxylin, fuchsin-ponceau and aniline blue) staining was carried out on 5-μm-thick cross sections.

### 2.5. Cell Isolation and Culture

**Figure 3:**
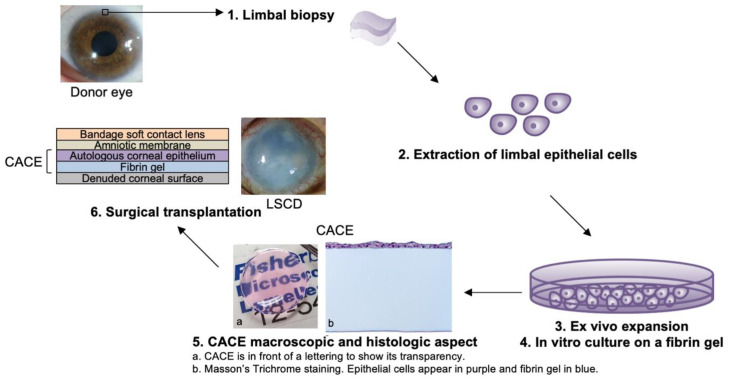
Schematic representation of the procedure. From a small limbal biopsy, epithelial stem cells were isolated. Cells were expanded ex vivo, seeded on a fibrin gel and cultured until CACE was ready to implant on the denuded corneal surface of the patient. The CACE is protected with a sutured human amniotic membrane and a bandage soft contact lens.

Figure 3 shows a schematic representation of the experimental procedure, from the cell biopsy to the surgical transplantation of the CACE.

Autologous limbal stem cells were harvested under local anesthesia, performing a 3 mm^2^ biopsy in the superior limbal quadrant of the contralateral cornea. Following appropriate eye anesthesia and measures to limit the risks of infection, a cornea limbal segment measuring about 3 mm lengthwise started at 1 mm from the limbus in the clear cornea using a circular blade (Crescent Blade) was dissected. The dissection was completed on the conjunctival side using Bonn forceps and a cornea scissor.

**Figure 4:**
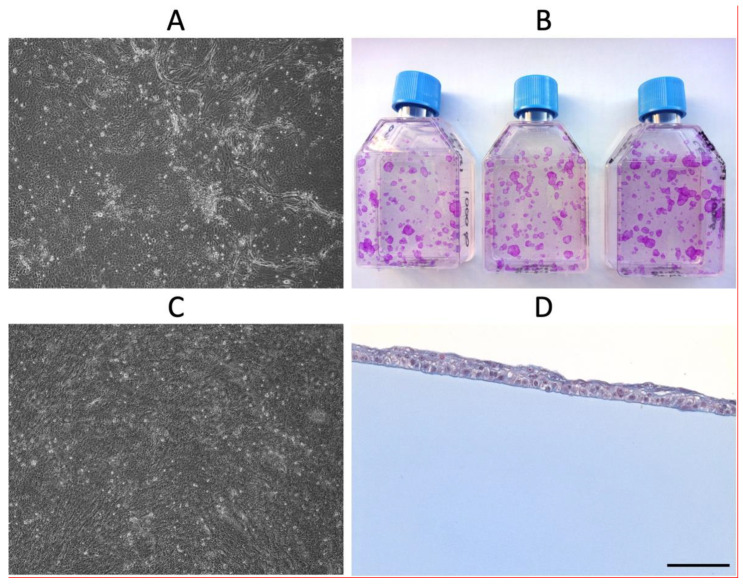
Identification of corneal epithelial cells. After each cell isolation (**A**) and once seeded on the fibrin gel (**C**), the typical epithelial cell morphology is confirmed under a phase contrast microscope. (**B**) Their ability to proliferate is evaluated using the clonogenic assay. Clones are stained with 2% rhodamine. (**D**) The formation of a non-keratinized stratified epithelium is confirmed by histological analysis of CACE samples. (**A**,**C**): 4× magnification. (**D**): Masson’s trichrome staining (scale bar: 100 µm).

The biopsy was quickly placed in a sterile container filled with cold transport medium (90% DMEM (VWR, Mississauga, ON, Canada), 10% fetal bovine serum (FBS, Seradigm, South Logan, UT, USA), 90 UI/mL Penicillin G (Fresenius Kabi, Toronto, ON, Canada), 22.5 µg/mL Gentamicin (MP biomedicals, Solon, OH, USA), 0.45 µg/mL Fungizone (Bristol-Myers Squibb Canada, St-Laurent, QC, Canada)) and then transported to the Laboratoire d’organogenèse expérimentale (LOEX) of the CHU de Québec-Université Laval in Quebec city. If the participant was allergic to antibiotics, the transport medium contained 27 µg/mL Vancomycin (Fresenius Kabi) and 22.5 µg/mL Gentamicin. The isolation of limbal stem cells and the subsequent culture were performed as previously described [19]. The delay between the limbal biopsy harvesting and the corneal epithelial cell’s extraction ranged between 52 and 163 min (mean 93 min, standard deviation (SD) = 35 min, n = 14). Briefly, the limbus was incubated overnight at 4 °C in a 2.5 mg/mL solution of Dispase II (Roche Diagnostics, Laval, QC, Canada) in 0.01M HEPES (Mallingckrodt, Phillipsburg, NJ, USA), 1 mM CaCl_2_. Using a dissection microscope and tweezers, the epithelium was separated from the stroma and placed in preheated 0.05% Trypsin (Life Technologies, Grand Island, NY, USA) 0.01% EDTA (J.T. Baker, Center Valley, PA, USA) solution and incubated for 15 min at 37 °C. Epithelial cell medium (DMEM:Ham’s F12 (VWR, Mississauga, ON, Canada) (3:1), 5% FetalClone II serum (Hyclone, South Logan, UT, USA), 5 µg/mL Insulin (SAFC, Lenexa, KS, USA), 0.4 µg/mL Hydrocortisone (Galenova, St-Hyacinthe, QC, Canada), 10^-6^ M Isoproterenol hydrochloride (Sandoz, Boucherville, QC, Canada), 10 ng/mL Epidermal Growth Factor (R&D System Quality, Minneapolis, MN, USA), 100 UI/mL Penicillin G, 25 µg/mL Gentamicin) was then added in order to obtain a medium:trypsin ratio of 1:1 and centrifuged. The pellet was resuspended in an epithelial cell medium. The corneal epithelial cells were subsequently seeded on a feeder layer of irradiated human fibroblasts (iHFLs). Cells were regularly observed with phase contrast microscopy to ensure their epithelial aspect and to follow their growth (Figure 4A). A fibrin gel (Tisseel^®^ kit VH/SD, Baxter Hyland Immuno, Mississauga, Ont., Canada) was prepared as follows: the thrombin solution of the kit was diluted in a saline solution of 1% (*w*/*v*) NaCl and 1 mM CaCl_2_ for a final concentration of 2.7 KUI/mL. The protein solution of the kit was diluted in a saline solution of 2% (*w*/*v*) NaCl and 1.86 mm CaCl_2_ for a final concentration of 43.06 mg/mL. Both solutions were mixed at a ratio of 1:1 and poured as quickly as possible in a 3-cm-diameter Teflon ring placed in a bacteriological petri dish of 60 mm surrounded by an 1.5% (*p*/*v*) Agarose (JT baker) gel. The fibrin gel was obtained after a polymerization step at 37 °C for a few hours. After 6 to 11 days (mean 8.9 d, SD = 1.6, n = 15), the epithelial cells reached between 60 and 95% subconfluence (mean 83.8%, SD = 10.3%, n = 15); they were trypsinized and seeded on fibrin gel at a cell density of 4000 to 6000 cells/cm^2^ and cultured in epithelial cell medium. The serine protease inhibitor aprotinin (130 KIU/mL, Nordic Pharma, Theale, Reading, UK) was added to the culture medium to prevent degradation of the fibrin gels. In parallel, the ability of epithelial cells to proliferate was evaluated using a clonogenic assay; they were seeded on iHFL at a cell density of 40 cells/cm^2^. After 9 days, colonies were fixed with 3.7% formol and stained with 2% rhodamine (Figure 4B). The growth of cells cultured on the fibrin gel was followed by phase contrast microscopy (Figure 4C). The corneal epithelial cells reached 100% confluence on the fibrin gel between 3 and 5.8 days (mean 4.6 d, SD = 0.7 d, n = 15). The tissue was either prepared for grafting (n = 3) or further cultured for a maximum of 2.5 days (mean 1.7 d, SD = 0.5 d, n = 12). The samples were processed for histological analysis (Figure 4D). The total production time therefore ranged from 12 to 19 days (mean 14.9 d, SD = 0.7, n = 15). The final CACE graft was cut in the center using a 14 mm trepan. The graft was then safely transferred into a sterile contact lens container containing transport medium and epithelium side-up. The grafting procedure was performed within 4 h.

### 2.6. Surgical Procedure

Prepping was done with drops of 0.5% proparacaine and 5% povidone-iodine instilled in the conjunctival fornix. The patients were either under general or local anesthesia. Local anesthesia was performed using a sub-tenon injection through a transconjunctival route into the lower temporal quadrant with approximately 3 mL of an anesthetic solution composed of 2% Xylocaine without epinephrine and 0.5% Marcaine 1:1 using a blunt-tipped metallic cannula. A 2% Xylocaine gel was then applied to cover the ocular surface. Fibrovascular membranes, conjunctivalization and abnormal epithelial cells were removed by gentle debridement. 

The fibrin gel covered with the CACE graft was placed on the denuded corneal surface and fixed with four 10–0 Vicryl^®^ sutures. It was then protected with a human amniotic membrane sutured with eight 10–0 Vicryl^®^ sutures and finally covered with a bandage soft contact lens. A solution of Moxifloxacin 0.5% and 1% prednisolone acetate was applied and a semi-compressive bandage was put in place.

Following grafting, an appropriate regime of anti-inflammatory and prophylactic antibiotic treatment was given. The bandage soft contact lens was left in place to allow time for the CACE to integrate into the surface of the cornea (about 2 to 4 weeks). The amniotic membrane was left in place until dissolution, except in cases where the amniotic membrane became dehiscent and irritated the ocular surface, where it was partially debrided.

### 2.7. Follow-Up and Outcome Assessment

Follow-up evaluations were planned the day after surgery, then every week for one month and monthly afterwards until 1 year had passed. After one year, the evaluations could be distanced in time according to the judgment of the ophthalmologist. The clinical assessment and BCVA of the treated eye were based on the same criteria as for the baseline evaluation. They were used to estimate anatomic and functional outcomes. A one-year follow-up was completed for all participants. Eleven participants completed 3 years of follow-up and nine patients reached five years.

The graft was considered an anatomical success when there was overall improvement in the ocular surface, as assessed by anatomic scores. It was considered an anatomical failure if the ocular surface showed no improvement. Functional success was achieved when the final BCVA was improved of ≥2 lines on the Snellen chart if BCVA at baseline was 6/120 or better. If patients improved by 1 line, it was considered a partial success. In cases where the initial BCVA was not measurable (i.e., worse than 6/120), final BCVA needed to be 6/60 or better in order to be classified as a success. Attaining 6/120 at the last follow-up resulted in partial success. Failure was defined as no improvement or worsening of BCVA. 

### 2.8. Statistics

Clinical assessment of anatomic scores and BCVA before the graft were compared with results at 1-, 3- and 5-years post-op. Since there was no control group, proportion ratios were expressed as percentages with a binomial proportion confidence interval of 95% (CI95). For the anatomical scores, the difference in the mean score between baseline and post-operative times was assessed using a paired sample t-test. All statistical analyses were performed using R software. The functional success rate observed was compared to what has been reported in the scientific literature [26]. The two-sided p-value was determined by exact calculations based on the binomial distribution. The significance threshold was set at 0.05.

## 3. Results

### 3.1. Study Population

Fifteen patients with unilateral LSCD were grafted with CACE. Two patients required a second CACE graft at five years of follow-up, as LSCD recurred (patient 3 and 9). Etiologies of LSCD varied greatly: three patients developed LSCD after years of topical glaucoma treatment; two after chemical burns; three had idiopathic LSCD; two had LSCD as a result of multiple surgeries; one patient had LSCD following an ocular surface squamous neoplasia excision; one after ocular radiotherapy treatments for a mucous membrane lymphoma; one after Interferon and Mitomycin treatment for an ocular surface squamous neoplasia; one following CO_2_ laser treatment for a non-specified limbal malignant tumor; one secondary to mycosis fungoides. The mean age of the patients was 54.6 years old and 40% were men (see Table 1). Patients who were biopsied were all diagnosed with LSCD by a pathologist before entering the study.

### 3.2. Adverse Events

The list of main adverse events observed after CACE grafting and other procedures performed later than one year after CACE grafting are presented in Table 2 and Table 3, respectively. No severe adverse events (SAE) due to CACE were observed. Most adverse events (AE) were minor in severity and rarely related to the research product. Therefore, CACE was considered a safe product.

### 3.3. Baseline Evaluation

At baseline, the mean scores for corneal opacity were 1.9 and 1.2 for central vascularization, as assessed by slit-lamp examination (Table 1). Every patient had tried medical management of LSCD without success prior to enrollment. Ten patients (66%) had visual acuity worse than 6/60.

### 3.4. Anatomical Outcomes

**Figure 5:**
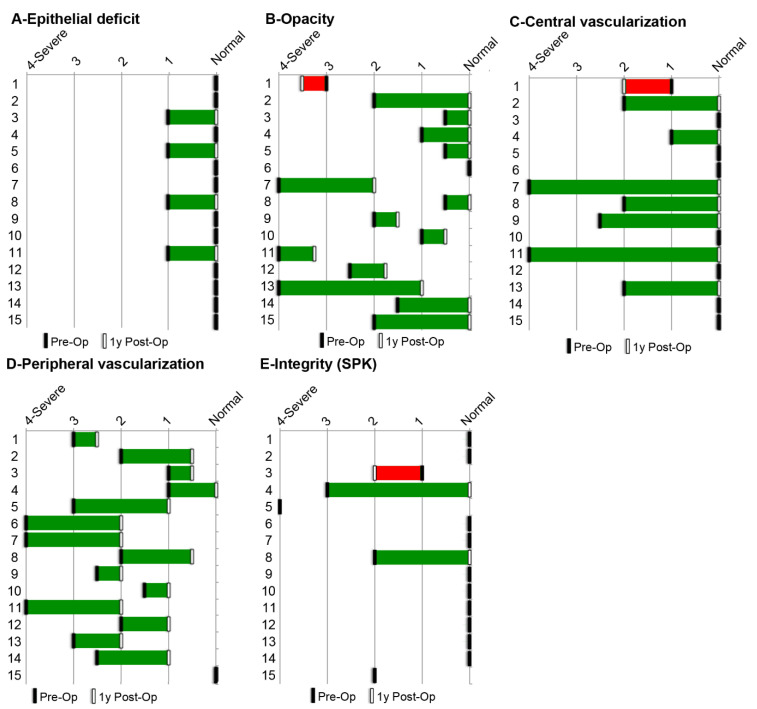
Anatomical assessment of each patient. The clinical assessment at baseline (Pre-Op) and 1 year after CACE grafting (Post-Op) was based on slip lamp examination and scoring from 0 (normal) to 4 (highest severity level). Epithelial defect, determined with fluorescein staining; corneal opacity; peripheral vascularization; central vascularization; and integrity of the ocular surface, determined by the presence of superficial punctate keratitis (SPK), are presented for the anatomical assessment.

The grafts were performed from 2013 to 2018. The mean follow-up period was 51.3 months. Data were collected at each visit. The results obtained at one year for each assessed LSCD-associated symptom are presented in Table 4 and Figure 5. The anatomical outcomes improved for all patients except 1 (93% [CI95: 68–100%]) at one year after grafting. Epithelial defects of various etiologies, present in four participants, improved in all patients (100% [CI95: 40–100%]). Significant corneal opacities, present in 14 patients at the beginning of the study, improved in 13 of them (93% [CI95: 66–100%]). Central vascularization, present in 8 patients at the beginning of the study, improved in 7 patients (88%, [CI95: 47–100%]). Peripheral vascularization, present in 14 patients at the beginning of the study, improved in all patients (100% [CI95: 77–100%]). Ocular surface integrity, evaluated by the presence of superficial punctate keratitis (SPK), initially compromised in 5 patients, improved in 2 patients (40% [CI95: 5–85%]). Considering that corneal opacity and central vascularization have a great impact on vision clarity, we believe that success was achieved for the majority of patients after one year. Furthermore, a clear cornea was maintained at the last recorded follow-up in 87% [CI95: 60–98%] of patients (see Table 4). Globally, mean score improvement for corneal opacity and central vascularization was observed at 1, 3 and 5 post-operative years (*p* < 0.05) (Table 4). 

### 3.5. Functional Outcomes

The functional outcome was evaluated using the best-corrected visual acuity score after one year. Since no other surgery was performed during the first year after CACE grafting, the functional outcome was attributed to the CACE graft. Overall, the functional outcome of the procedure was considered a success in seven patients (47% [CI95: 21–73%]), a partial success in 1 patient (7% [CI95: 0–32%]) and a failure in 7 patients (47% [CI95: 21–73%]). Compared to the 69% functional success rate reported for SLET in the systematic review of Shanbhag et al. [26], the p-value associated with our observed success rate of 47% is *p* = 0.089, indicating that the difference is not significant. Considering that the best corrected visual acuity depends on the quality of the epithelium but also on the adequate structure of the stroma and functionality of the endothelium, 10 patients had additional surgeries after the one-year follow-up.

### 3.6. Causes for Poor Visual Recovery

Patient 3 was a patient requiring a second CACE graft because of a recurrence of LSCD many years after the initial CACE graft. For this patient, despite anatomical success after the second CACE graft, the functional outcome was considered poor because of a decrease in BCVA. This decrease was associated with an epiretinal membrane and was not related to the ocular surface.

Similarly, patient 6 developed bacterial keratitis 1 year after CACE grafting, resulting in a decrease in BCVA secondary to an inferior corneal scar. However, the central cornea remained clear and untouched by signs of LSCD, indicating good anatomical results.

Patient 7 LSCD eye was deeply amblyopic because of an early age severe conjunctivalization of most of the corneal surface. Her affected eye was also highly myopic and it was left aphakic after cataract surgery 3 years after the CACE graft was performed. Both amblyopia and aphakia are responsible for preventing the improvement of vision, despite a clear corneal surface.

**Figure 6:**
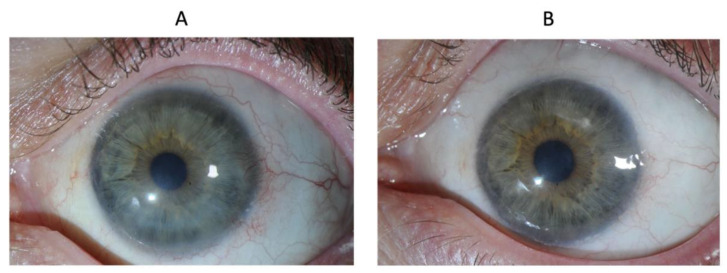
Eye of patient 8 at baseline (**A**) and at 1 year (**B**).

Patient 8 was left with a central scar from the initial chemical burn. Opacity and vascularization scores were zero at the last follow-up, indicating good anatomical results. However, vision was not restored due to stromal opacification (Figure 6).

**Figure 7:**
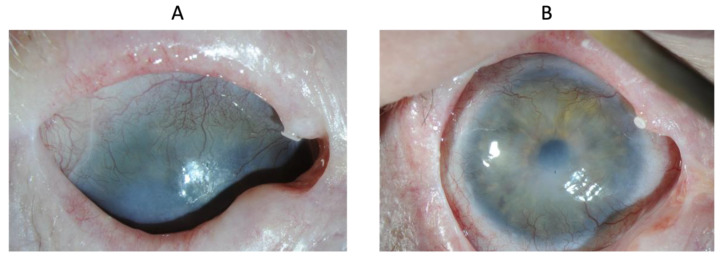
Eye of patient 9 at baseline (**A**) and at two years (**B**).

Patient 9 received localized radiotherapy for conjunctival lymphoma, which is a treatment that could cause severe tissue alterations detrimental to ocular surface regenerative properties [27]. Patient 9 was a patient requiring a second CACE graft because of a recurrence of LSCD many years after the initial CACE graft. Consequently, an improvement in best visual acuity from hand movement to 6/120 is significant, more so when considering the absence of LSCD signs at the last visit (Figure 7).

**Figure 8:**
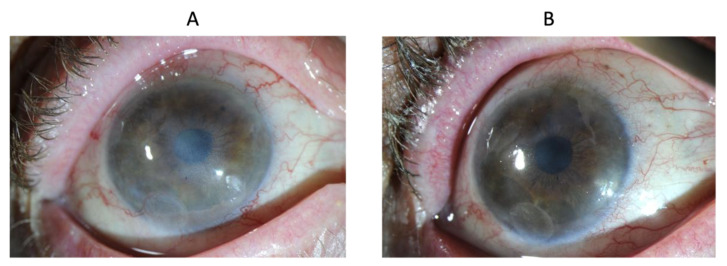
Eye of patient 10 at baseline (**A**) and at one year (**B**).

Patient 10 had an epithelioma (CIN: conjunctival intraepithelial neoplasia) excised and was treated with interferon and mitomycin C. BCVA one year after the CACE graft improved significantly (Figure 8).

**Figure 9:**
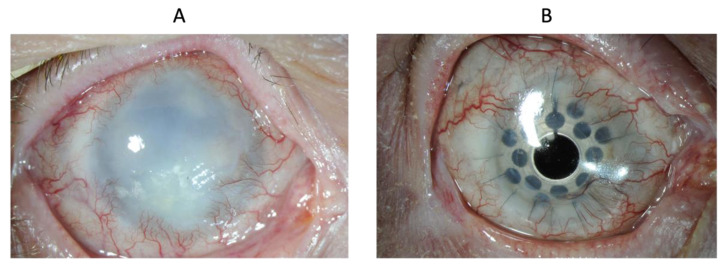
Eye of patient 11 at baseline (**A**) and at three years (**B**).

Patient 11 suffered a thermal burn after the use of a CO_2_ laser to treat an ocular surface squamous neoplasia. Two years after CACE grafting, the clarity of the corneal stroma was restored with penetrating keratoplasty. A few months later, a tractional retinal detachment with cyclitic membrane required a pars plana vitrectomy with retinopexy, endolaser and intravitreal silicone oil. The patient received a Boston keratoprosthesis as a protective measure against the development of silicone oil-induced keratopathy (Figure 9). Hence, the final BCVA of light perception (LP) is the result of retinal complications and not of ocular surface disorders.

## 4. Discussion

Our multidisciplinary team of scientists, clinicians and ethical/legal experts conducted the first clinical trial in Canada to offer CACE as a personalized treatment for unilateral LSCD. This monocenter trial was conducted at the CHU de Québec-Université Laval Hospital, where 15 patients were enrolled and followed for one to five years. The findings of this study suggest that CACE is a safe and promising treatment for unilateral LSCD for the healing of the corneal surface and restoration of visual acuity. The CACE graft was well tolerated by every patient and no severe adverse events were noted during the follow-up period (mean follow-up 4.3 years). Combined functional success and partial success rates with inclusion of BCVA were 53% [CI95: 27–79%]. This result is comparable to those reported in the literature [26]. However, given the small number of patients, our estimation of the success rate of CACE in treating LSCD lacks precision so far. Our team recently received approval from regulatory authorities to increase the number of patients in the clinical trial.

When the study was originally designed, we feared that epithelial and stem cell implantation would be jeopardized if not adequately protected from the mechanical effect of the natural blinking process. This is why an amniotic membrane was sewn over the CACE graft. Previous studies have included mostly burn-dependent limbal stem cell deficiency patients. These injuries usually occur in younger individuals without other significant ocular surface impairments. Many of the patients included in our study also suffered from major ocular surface-related problems: lid abnormalities and scarring, important Meibomian gland dysfunction, conjunctival scarring, etc. Despite our best efforts, these comorbidity factors were probably at least partially responsible for poorer results in severely damaged ocular surface patients, especially post-radiotherapy.

Various factors could have influenced our success rate, including the etiology of LSCD, the definition of success and the lack of statistical power as aforementioned. Many studies have focused primarily on LSCD following chemical burns, which tend to respond better to CLET when compared to some other etiologies [28]. Our study included severe cases of LSCD, all confirmed either by limbal biopsy or by obvious causes. Etiologies included chemical and thermal burns, radiation therapy, tumors and neoplasia, glaucoma medication, repeated surgeries and idiopathic cases. Comparison of CLET results from studies of other groups is difficult, as the definition of success varies greatly from improvement of corneal conjunctivalization to restoration of a clear corneal epithelium [28,29,30]. Visual acuity might not be the best criterion of success for CLET, as it can decrease for a multitude of reasons other than ocular surface disorders. Therefore, as CLET only corrects corneal surface defects, including visual acuity, our study was bound to result in lower success rates for functional assessment. Despite these issues, our results are concurrent with those of Haagdorens et al., who achieved a 55% improvement of two lines or more in BCVA after CLET in 2016 [31], and with the 69% improvement reported in the systematic review of Shanbhag et al. [26]. The safety of our technique was also demonstrated by the absence of complications in the donor eye and the benign nature of adverse events in the operated eyes.

When compared with other surgical alternatives to unilateral LSCD, such as SLET and CLAU, CLET has the advantage of requiring a smaller biopsy from the donor eye. This strength may be crucial in the choice of treatment in cases of bilateral LSCD, where every millimeter of limbus harvested for grafting or to initiate culture has the potential to induce secondary LSCD in the healthier eye. Furthermore, cell culture allows for quality control of every transplant, as well as the option of cryopreservation for later use of the cells as needed. CLET also has the potential to treat congenital causes of LSCD when combined with gene therapy.

However, in severe bilateral LSCD, where no biopsy can safely be performed, the limbal stem cell source is limited to allografts. Keratolimbal allograft (KLAL) uses limbal tissue from cadaveric eyes and requires long-term immunosuppressive treatments to prevent graft rejection [32]. To bypass this issue, researchers have explored other sources of autologous stem cells. In 2003, Nakamura et al. described cultured oral mucosal epithelial transplantation (COMET). The oral mucosa shares some physical properties and functions with the corneal epithelium, which makes it a usable substitute for LSCs. Results showed an ability to regenerate an avascular, stable and epithelialized corneal surface in patients with severe LSCD, although the outcome was poorer than with autologous LSC [33,34,35]. Other stem cell sources have been proposed (for a review, see Miotti et al., 2021 [36]). They include the use of induced pluripotent stem cells derived from hair follicles or dermal fibroblasts to create corneal organoids in 3D culture systems that show interesting in vitro results [37]. Mesenchymal stem cells (MSCs) extracted from bone marrow have also been studied in animals, where they have demonstrated their safety and ability to restore the corneal surface in rabbits with LSCD [38]. Umbilical cord-derived MSCs have shown potential for differentiating into corneal epithelial cells in a 3D anterior cornea model [39]. Moreover, immature dental pulp and oral mucosa have been reported to be favorable sources of mesenchymal stem cells for treating LSCD [34,40]. These initial studies remain to be confirmed by large clinical trials in humans.

Although the functional success rate, in terms of visual acuity, in our cohort following CACE grafting initially appears lower than in the literature, it is mostly the result of the inclusion of BCVA as a criterion for success. It is obvious that BCVA can vary greatly from one visit to another and can decrease with ocular conditions unrelated to the ocular surface. In our cohort, this was the case in 47% of patients. As presented in the results, these patients had severe ocular conditions that did not affect only the epithelium. CACE only corrects the corneal epithelium by providing limbal stem cells. When the stroma is also affected, the BCVA can be diminished, even though the CACE graft is successful. 

Radiotherapy is often part of the treatment plan for cancer. However, one of its main side effects is the permanent alteration of irradiated tissues. We believe this effect might explain, to a certain point, the difficulty for the CACE graft to heal adequately over such altered ocular surfaces. It is well known in the skin that radiotherapy induces chronic wounds that are very difficult to heal [41].

When success is solely defined as the disappearance of LSCD signs based on anatomic assessment (epitheliopathy and conjunctivalization), only one patient did not achieve this objective, resulting in a true success rate of 93% [95CI: 68–100%]. Moreover, all but one patient had a major improvement of corneal surface post-op, which was maintained at the last follow-up, supporting the efficacy of CLET on long-term preservation of limbal stem cells. Additionally, 87% [CI95: 60–98%] of patients had attained corneal clarity with an opacity and a central vascularization score of zero by the last recorded follow-up.

## 5. Conclusions

Our study is among the first to report CLET-based technology results in severe unilateral LSCD caused by radiation therapy, tumors and neoplasia, which are known to respond poorly to ocular surface treatments. Outcomes demonstrate the safety of CACE via the absence of major adverse events and are promising for improving LSCD-associated symptoms. BCVA, although a useful indicator of functionality, is not a good indicator for the success of a treatment focused on the limbal/corneal epithelium as it may vary independently of the corneal surface and thus wrongly influence success rates. Prolonged follow-up illustrates the ability of CLET to restore and maintain a clear cornea over time. Complementary studies with a greater number of patients are required to further assess the long-term efficacy of this technique.

## Figures and Tables

**Table 1:** Patient Characteristics.

Patients	Age	Sex	Cause of LSCD	Baseline VA	Baseline Corneal Opacity Score	Baseline Central Vascularization Score	Follow-Up Time (Months)
**1**	37	M	Chemical burn	HM	3	1	81.3
**2**	18	F	Idiopathic	FC	2	2	24.7
**3**	83	F	Glaucoma medication	HM	0.5	0	71.0
**4**	69	M	Tumor excision	6/15	1	1	74.8
**5**	38	F	Idiopathic	FC	0.5	0	62.4
**6**	80	M	Glaucoma medication	6/15+1	0	0	40.9
**7**	32	F	Idiopathic	LP	4	4	59.6
**8**	33	M	Chemical burn	6/9-2	0.5	2	56.8
**9**	64	F	Radiotherapy for mucosa associated lymphoid tumor	HM	2	2.5	52.9
**10**	70	M	CIN (conjunctival intraepithelial neoplasia) Interferon and Mitomycin treatment	FC	1	0	46.3
**11**	79	F	CIN (conjunctival intraepithelial neoplasia) CO_2_ laser treatment	LP	4	4	64.2
**12**	40	F	Glaucoma medication and surgeries	FC	2.5	0	55.5
**13**	57	M	Mycosis fungoides	FC	4	2	19.1
**14**	51	F	Chemical burn	6/15+2	1.5	0	16.8
**15**	68	F	Multiple surgeries	6/18+2	2	0	43.9

LSCD—Limbal Stem Cell Deficiency, VA—Visual Acuity, MALT—Mucosa associated lymphoid tumor, CI—Conjunctival intraepithelial neoplasia.

**Table 2:** Adverse events one year after the CACE graft ^1^.

	1. Chemical Burn	2. Idiopathic	3. Glaucoma Medication	4. Tumor Excision	5. Idiopathic	6. Glaucoma Medication	7. Idiopathic	8. Chemical Burn	9. Radiotherapy for MALT Lymphoma	10. CIN Interferon and Mitomycin	11. CIN CO_2_ Laser	12. Glaucoma Medication and Surgeries	13. Mycosis Fungoides	14. Chemical Burn	15. Multiple Surgeries
**BCL related event**	1		1				4	1		2			1		2
**Superficial punctate keratitis**			1												
**Epithelial defect**	2		3			1	1	1	4	2	1	2	2	1	
**Epithelial irregularity**			1	3		1		2							1
**Eyelash trichiasis**							1		1						
**Ocular surface disorder**		1		2	3					1	1				
**Amniotic membrane related event**				1						1			1		
**Corneal surface infection**					2	2			1						1
**Eyelid cellulitis**															1
**Corneal opacity**		1									1				
**Descemetocele without leak**													1		
**Glaucoma**															1
**Post-operative hemorrhage**	1														
**Pseudopterygium**							1								
**Symblepharon recurrence**													3		
**Fall**									1						
**Shock: blow with forceps**											1				
**Number of events**	**4**	**2**	**6**	**6**	**5**	**4**	**7**	**4**	**7**	**6**	**4**	**2**	**8**	**1**	**6**

^1^ Most adverse events (AE) were minor in severity and rarely related to the research product. BC—Bandage contact lens. MALT = Mucosa-associated lymphoid tumor. CIN = Conjunctival intraepithelial neoplasia.

**Table 3:** Procedures performed more than one year after CACE grafting ^1.^

	1. Chemical Burn	2. Idiopathic	3. Glaucoma Medication	4. Extensive Limbal Dysplasia Excision	5. Idiopathic	6. Glaucoma Medication	7. Idiopathic	8. Chemical Burn	9. Radiotherapy for MALT Lymphoma	10. CIN Interferon and Mitomycin	11. CIN CO_2_ Laser	12. Glaucoma Medication and Surgeries	13. Mycosis Fungoides	14. Chemical Burn	15. Multiple Surgeries
**Cataract surgery**							1			1				1	1
**Lamellar Keratoplasty**							1					1	1		
**Superficial Keratectomy**				1					1						1
**DSAEK**												1			
**Penetrating keratoplasty**											1			1	
**Laser iridotomy**															1
**Retina reattachment**											1				
**Boston Keratoprosthesis**											11				
**Repeat CACE**			1						1						
**Tarsorrhaphy**									1						
**Lid taping**							1								
**Number of procedures**	0	0	1	1	0	0	3	0	3	1	3	2	1	2	3

^1^ See results section for more details. MALT = Mucosa-associated lymphoid tumor. CIN = Conjunctival intraepithelial neoplasia. DSAEK = Descemet Stripping Automated Endothelial Keratoplasty.

**Table 4:** Anatomical and functional assessment of patients at 1, 3 and 5 years of follow-up.

Patients	Corneal Opacity ^1^				Central Vascularization ^1^				Anatomical Outcome ^3^	BCVA ^2^			Functional Outcome ^4^
	Pre-op	1y	3y	5y	Pre-Op	1y	3y	5y		Pre-op	1y post-Op	Longest FU	
**1**	3	3.5	3	0	1	2	2	0	F	HM	HM	6/45	F
**2**	2	0			2	0			S	FC 30 cm	6/24	6/9	S
**3a ^6^**	0.5	0	1.5	1.5	0	0	0	0	S	HM	6/45	6/45	S
**3b ^6^**	1.5	0	ND ^5^		0	0	ND ^5^		S	6/45	6/120	6/120	F
**4**	1	0	0	0	1	0	0	0	S	6/15	6/6	6/7.5	S
**5**	0.5	0	0	0	0	0	0	0	S	CF 60 cm	6/18	6/24	S
**6**	0	0	0		0	0	0		S	6/15+1	6/45	6/60	F
**7**	4	2	2	0	4	0	0	0	S	LP	6/120	HM	PS
**8**	0.5	0	0	0.5	2	0	0	0	S	6/9-2	6/18	6/9	F
**9**	2	1.5	2	0	2.5	0	0	0	S	HM	CF 1 m	6/120	F
**10**	1	0.5	0		0	0	0		S	CF	6/21	6/18	S
**11**	4	3.25	0	0	4	0	0	0	S	LP	LP	LP	F
**12**	2.5	1.75	0	0	0	0	0	0	S	CF	6/45	6/18	S
**13**	4	1			2	0			S	CF	6/60	6/60	S
**14**	1.5	0			0	0			S	6/15+2	6/45	6/12	F
**15**	2	0	0		0	0	0		S	6/18+2	6/30	6/30	F
**Mean Score ^7^**	**1.9**	**0.7**	**0.7**	**0.1**	**1.2**	**0.1**	**0.2**	**0.0**					
**Mean Score Improvement ^8^**		1.2 *	1.2 *	1.8 *		1.1 *	**1.0**	1.2 *					

^1^ For corneal opacity and corneal vascularization a smaller score is better. ^2^ Functional score was assessed with the Best Corrected Visual Acuity (BCVA). ^3^ Anatomical outcome 1 year after grafting was assessed as described in Methods. ^4^ Functional outcome 1 year after grafting was assessed as described in Methods. ^5^ ND The follow-up was interrupted because of the death of patient 3, which was secondary to a systemic illness unrelated to the research product. ^6^ This patient required a second graft at five years of follow-up, as LSCD recurred. 3a: Results for the first CACE graft. 3b: Results for the second CACE graft. ^7^ For the calculation of mean scores, observation 3a was considered at baseline (pre-op), while for post-op results, it was 3b. ^8^ Mean score improvement = |mean score pre-op-mean score post-op|. * *p* < 0.05 (bilateral Student T test for paired sample). BCVA-Best corrected visual acuity, F-Failure, FU-Follow-up, y-year(s), CF-Counting fingers, HM-Hand movement, LP-Light perception, cm-Centimeters, m-Meters, PS-Partial success, S-Success.

## Data Availability

Not applicable.
